# Food Economy in Institutions

**Published:** 1916-02-19

**Authors:** 


### FOOD ECONOMY IN INSTITUTIONS.

### How to Prepare a Menu.

THE call for national economy has brought many writers into the field who formerly left the housekeeper's domain sternly alone. It is to be regretted that the more scientific principles of catering have so long lain outside the attention of the men and women who have the practical side to attend to, for when science lends its aid to economy, cheaper and better meals ought to be the result. "Food Economy in War Time" is a little pamphlet written by Professors Wood and Hopkins, the former Drapers Professor of Agriculture, the latter Professor of Biochemistry. Laying their heads together they have presented in popular form the results of many valuable researches, new and old, into the constituents and comparative cost of common articles of food. A flood of light is thrown by their experiments on the economical preparation of a menu, and we venture to assert that were its lessons taken to heart both families and institutions might effect reductions of from 25 to 50 per cent. in the average cost of the food.

The writers adopt the usual estimate that about 4 oz. of protein are required per head per diem for the growth and repair of the body, and some 3,000 calories to meet its requirements in respect of energy. These estimates are for persons occupied in "light muscular work," including professional men, and presumably professional women. To test the quantities of food necessary to meet these requirements has always proved a stumbling-block for those who wish to reduce principles to practice, so remote is the scale of science from the butcher's and baker's bill. Messrs. Wood and Hopkins have undertaken to bridge the gulf, and with the aid of their tables the housekeeper can now determine without trouble how much protein per head, how many calories of energy she serves to her diners, and at what cost. The results are somewhat surprising, as a few examples taken from the tables show. In the form of fresh butcher's meat a pound of protein costs from 5s. to 8s. In the form of fowl it rises to 8s. 4d., and of chicken to 13s. 4d. per lb. The cost of a pound of protein in fish varies from 2s. 7d. in herrings to 12s. in hake. In fresh eggs the protein works out at 7s. per lb., reckoning the cost of each egg only at 1½d. The cost of protein works out in bread at 5½d. per lb., in milk at 2s. 4½d. per lb., in cheese at 2s. 6d. per lb., in lentils at 10d. per lb., in oatmeal at 3d. per lb.

To ensure that the food actually put on the table contains the proper constituents in the amount necessary to keep each individual up to the mark as regards energy and repair, columns are given which state the weight of protein and the number of calories present in each pound of the articles quoted. By the aid of these figures the food value of any ordinary allowance may be readily tested. The following common daily allowance for the institution, as tested by the tables, gives an interesting result:—

| | Cost d. | Protein oz. | Calories |
|---|---|---|---|
| Bread, ½ lb. ... ... ... | 1 | ¾ | 612 |
| Meat and ham, ½ lb. ... ... | 7 | 2½ | 450 |
| Milk, 1 pt. ... ... ... | 2 | ¾ | 406 |
| Butter, 2 oz. ... ... ... | 1½ | — | 450 |
| Jam, 2 oz. ... ... ... | ½ | — | 175 |
| Potatoes, ½ lb. ... ... ... | ¼ | ⅛ | 155 |
| Sugar and sweet pudding (say) | 1 | ⅛ | 150 |
| | 1 1¼ | 4¼ | 2,398 |

As it stands, the diet is ample as regards protein, but deficient in calories. Were the milk reduced, as often happens, to a quarter of the quantity, it would fall below the level in both directions. To reduce the cost and add to its nutritive properties the quantity of meat could be reduced by one-fourth, and its place supplied by some appetising supplementary dish such as savoury rice, curried lentils, or boiled beans with onion, at the mid-day meal. At breakfast the dish of ham or eggs could be replaced on alternate days by oatmeal porridge or hot oat-cake, and for supper milk pudding, macaroni, or vegetable soup could be substituted for cold meat. Both in protein and calories the proposed alterations would show an improved result.

It has been pointed out that national economy requires every one to eat two pounds of meat less per month, or an average of two ounces less a day. In an institution boarding a hundred persons this would mean a reduction of at least £10 in the butcher's bill. In order to afford the best opportunity to the diners of changing their diet for the better there should be no sudden revolution in the menu. Extra meat-free dishes should be handed round and the size of the meat helping reduced, though every facility should be afforded for obtaining, if desired, another helping of meat.

